# Effects of Gamma Radiation on the Sterility Assurance, Antibacterial Ability, and Biocompatibility of Impregnated Hydrogel Macrosphere Protein and Drug Release

**DOI:** 10.3390/polym13060938

**Published:** 2021-03-18

**Authors:** Po-Sung Fu, Jen-Chyan Wang, Pei-Ling Lai, Shih-Ming Liu, Ya-Shun Chen, Wen-Cheng Chen, Chun-Cheng Hung

**Affiliations:** 1 School of Dentistry, College of Dental Medicine, Kaohsiung Medical University, Kaohsiung 807378, Taiwan; posung.elegant@msa.hinet.net (P.-S.F.); jechwz@kmu.edu.tw (J.-C.W.); 2 Department of Dentistry, Kaohsiung Municipal Ta-Tung Hospital, Kaohsiung 80145, Taiwan; 3 Division of Prosthodontics, Department of Dentistry, Kaohsiung Medical University Hospital, Kaohsiung 807378, Taiwan; casting0118@gmail.com; 4 Dental Medical Devices and Materials Research Center, College of Dental Medicine, Kaohsiung Medical University, Kaohsiung 807378, Taiwan; 5 Advanced Medical Devices and Composites Laboratory, Department of Fiber and Composite Materials, Feng Chia University, Taichung 40724, Taiwan; 0203home@gmail.com (S.-M.L.); yaschen@fcu.edu.tw (Y.-S.C.)

**Keywords:** hydrogels, crosslinking, degradable, gamma (γ)-irradiation, sterilization, sterility assurance, antibacterial ability

## Abstract

Devices and medicines used in the medical field must be sterile. Gamma (γ)-irradiation is commonly used for sterilization because its high rate of penetration ensures uniform sterilization. To confirm that hydrogel macrosphere carriers inherit excellent liquid absorption with no cytotoxicity after γ-irradiation sterilization, investigating whether the physiochemical properties of hydrogel macrospheres differ before and after sterilization is essential. The present study evaluated the influence of the recommended 25-kGy γ-irradiation dose on the physicochemical characteristics and in vitro release of bovine serum albumin and vancomycin (an antibiotic medication) from alginate/gelatin with a *w*/*w* ratio of 1/4 crosslinking gel macrospheres. Gel macrosphere properties before and after sterilization were compared according to optical and scanning electron microscopy, infrared spectroscopy analysis, the amino residual crosslinking index, water absorption, degradation, sterility assurance, in vitro drug release, antibacterial ability, and cytotoxicity. The crosslinking index was almost unchanged; however, the γ-irradiation caused in situ hydrogel debonding and recrosslinking, which led to a decrease in the water absorption and increase in the degradation rate of the macrospheres after immersion. The release of gel macrospheres carrying vancomycin did not significantly affect antibacterial ability or biocompatibility after γ-irradiation. Accordingly, we conclude that γ-irradiation is suitable for macrospherical formulation.

## 1. Introduction

Hydrogel scaffolds with three-dimensional (3D) structures are generally used to provide physical and structural support to cells because the composition of the hydrogel is similar to an extracellular matrix. Hydrogels consisting of microspheres (in micrometers) or macrospheres (in millimeters) possess highly interconnected pores, which are key to maximizing blood absorption to enhance cell growth [1,2,3,4]. Therefore, hydrogel microspheres or macrospheres can potentially alter the function or performance of an implant in terms of drug delivery, regenerative therapies in assisting native regeneration, and restorative therapy toward repairing tissue or organs. Accordingly, biodegradable or absorbable hydrogel microspheres or macrospheres are the most widely used shapes in wound dressings, tissue engineering, regenerative medicine, and drug delivery [5,6,7].

Hydrogel scaffolds are mainly fabricated using physical and chemical crosslinking methods [8,9]. Hydrogel scaffolds fabricated through physical crosslinking are mainly formed due to the interaction of environmental conditions with hydrogen bonds and proteins. The physical crosslinking process involves freeze-thawing [10,11], stereo complex formation [12,13,14], ionic interaction [15,16,17], and hydrogen bonding [18]. The chemical crosslinking process involves Schiff base formation [19], grafting [20,21], radical polymerization [22,23,24], the condensation reaction [25,26], and high-energy irradiation [27,28]. A chemically crosslinked hydrogel is mainly formed through free radical polymerization, chemical reaction, energy irradiation, and catalytic enzyme crosslinking [29,30,31].

In biomedicine, the applied forms of hydrogel microspheres or macrospheres are solid, semisolid, and liquid hydrogels. Solid hydrogels have a network structure of ions and covalent crosslinks, and inorganic metals or microparticles or nanoparticles can be added to hydrogel to increase its antibacterial ability and enhance its mechanical properties [32,33,34,35]. Due to their excellent adhesion properties, semisolid hydrogels are advantageous for prolonging drug delivery and improving drug availability [36,37,38]. Liquid hydrogels easily incorporate drugs, proteins, and cells and are suitable for wound dressing. Besides, liquid hydrogels can be used to administer medication as a controlled release dose through injection [39,40,41,42].

Medical equipment, devices, and drugs are most commonly sterilized using gamma (γ)-irradiation. The main advantage of γ-irradiation is its high level of penetration and isothermal characteristics, which can appropriately manage heat-sensitive materials include inorganic metal oxides, polymer blends, or liquid crystals. In addition, γ-irradiation ensures uniform sterilization; thus, the risk of microbial contamination can be avoided after products are packaged. Historically, the United States permitted a relatively high sterility assurance level (SAL) of 10^−3^ for items such as surgical drapes and gowns. However, in recognition of certain Pharmacopeia requirements, as of 1998, the Conformitè Europëenne (CE) marking requires a SAL of at least 10^−6^. Due to the number of microorganisms and the drug resistance of materials, the most common γ-irradiation dose is 25 kGy [43,44,45,46,47].

Sterilization can be achieved by a combination of heat, chemicals, irradiation, high pressure, and filtration like steam under pressure, dry heat, ultraviolet, radiation, gas vapor sterilants, chlorine dioxide gas, etc. However, for products that cannot be sterilized in the final containers, aseptic processing is necessary. Besides, natural polymers undergoing radioactive sterilization may cause energy transfer, which leads to covalent bond breakage and the generation of free radicals that can change the physicochemical properties and activities of materials, thereby producing toxic substances. Therefore, confirming whether toxic products are generated after newly developed medical products are sterilized using γ-irradiation is essential. According to our preliminary test, the basic chemical structure of hydrogels was changed greatly and showed less stability at radiation doses of 30 and 50 kGy than 25 kGy due to amide degradation by γ-irradiation.

The current study aimed to determine whether the use of 25 kGy of γ-irradiation to sterilize a relatively large amount of hydrogel macrospheres on a millimeter-scale prevents or reduces cytotoxicity or changes in physical properties in the context of hydrogel macrospheres impregnated with protein or drugs.

## 2. Materials and Methods

### 2.1. Raw Materials 

The raw materials used in this study were gelatin (80–100 Blooms [USP-NF, BP, Ph. Eur.] pure, pharmaceutical grade, PANREAC, EU), alginic acid sodium salt from brown algae (low viscosity, Sigma-Aldrich^®^, St Louis, MO, USA), *N*-(3-dimethylaminopropyl)-*N*’-ethylcarbodiimide hydrochloride (EDC, molecular weight 191.70 g/mole, Sigma-Aldrich^®^, St Louis, MO, USA), vancomycin (GENTLE PHARMA CO., LTD, Yunlin County, Taiwan), and sucrose (KATAYAMA CHEMICAL CO., LTD, Osaka, Japan).

### 2.2. Preparation of Gel Macrospheres

Porous spheres were fabricated through solvent casting and particulate leaching. The preparation procedures were based on previously described methods [48,49]. A colloidal suspension was prepared after heating to 50 °C and mixing sodium alginate with gelatin in a *w*/*w* ratio of 1:4 and suspending the mixture in 10 mL of deionized distilled (DD) water. The colloidal suspension was also mixed with saccharose particles in a particle:colloid ratio of 4 g:1 mL, which formed more than 70% of the interconnected pores within the gel macrospheres after particle leaching. After the colloid was evenly mixed, an autoinjector was used to control the discharge rate and produced fixed-volume macrospheres. The fixed-volume particles containing colloidal were added dropwise into the solution with cross-linkers of 1% EDC and 0.5% anhydrous calcium chloride at 4 °C. Subsequently, the gel macrospheres were removed and reimmersed in 1% EDC solution for 24 h to complete the crosslinking reaction. The crosslinked gel macrospheres were soaked in DD water for 3 h at 25 °C and were washed three times for leaching the particles. The macrospheres were then dried in a vacuum. Air pressure was reduced to approximately 26 μbar through lyophilization for 3 d.

### 2.3. Bovine Serum Albumin and Vancomycin Loading as Well as Release Measures

The porous macrospheres were immersed separately in 0.1% bovine serum albumin (BSA) and 25 mg/mL vancomycin solution to conduct protein and antibiotic loading at 4 °C for 24 h. The solution was frozen at −20 °C for 3 h and dried in a vacuum through lyophilization for 24 h to fabricate gel macrospheres with BSA and vancomycin. The designated group of macrospheres with vancomycin after irradiation was HyS-Van25.

The release rates of the macrospheres with BSA and vancomycin were investigated using DD water. BSA- and vancomycin-loaded macrospheres weighing 0.01 g were suspended in Eppendorf tubes with 1 mL of immersion solution. The release rates of BSA and vancomycin were determined after 5 and 22 days, respectively, in a water bath at 37 °C. Triplicate tests were used at each time point (*n* = 3). The immersion solutions were withdrawn at each time point. The amount of BSA released from the porous spheres was evaluated using a bicinchoninic acid (BCA) protein assay kit (Prod#23225; Thermo Fisher Scientific, Rockford, IL, USA) by measuring the optical density (OD) at a wavelength of 570 nm in an enzyme-linked immunosorbent assay (ELISA) macroplate reader (EZ Read 400, Biochrom, Cambridge, UK). To determine the content of vancomycin released by the spherical drug carrier, the OD of the amino acid in vancomycin was measured at a wavelength of 562 nm to compare the relative quantitative regression curves.

### 2.4. Sterilization by γ-Irradiation

The hydrogel macrospheres were sterilized using γ-radiation. The commonly used unit of absorbed radiation dose is the gray (Gy), which is equivalent to 1 joule of energy absorbed per kilogram of material. Hydrogel macrospheres (HyS-w/o γ) with protein BSA or antibiotic vancomycin and blank macrospheres were placed in 3-mL glass vials labeled and packed. The samples were irradiated using ^60^Co-γ as the radiation source at China Biotech Corporation, Taichung, Taiwan. According to United States Pharmacopeial Convention (USP) recommendations, a sterilized dose of 25 kGy was used. After γ-ray irradiation, the sterilized hydrogel microspheres of HyS-w/γ were subjected to a sterility assurance test and appearance confirmation before the samples could be used for further testing and analysis. The relevant tests are described subsequently.

### 2.5. Characterization of Gel Macrospheres after Sterilization 

#### 2.5.1. Morphological Observations

Dried and wetted porous macrospheres of HyS-w/γ and HyS-w/o γ were observed using an optical microscope (OM; Primotech, ZEISS, Oberkochen, Germany). The wetted macrospheres were first immersed in excess DD water and placed at 37 °C for 24 h. The water-containing macrospheres were then removed, rinsed with saturated sponges, and placed on a glass slide for observation. The cross-sectional morphology was then examined through scanning electron microscopy (SEM; S-3000 N, Hitachi, Tokyo, Japan) and compared with the OM images to investigate the pore changes and morphological characteristics of the macrospheres before and after sterilization.

#### 2.5.2. Changes in the Crosslinking Index after Radiation

The residual number of amino groups observed was approximately related to the crosslinking index of hydrogels. Accordingly, ninhydin (2,2-dihydroxy-1,3-indanedione) reagent (Sigma-Aldrich) was used to investigate the residual amino groups of macrospheres before and after sterilization. Ninhydin reagent can react with amines and amino acids during colorimetric assays. Residual amino measurements were conducted according to the manufacturer’s instructions, and the OD was measured at a wavelength of 570 nm. The crosslinking index of the macrospheres before and after radiation was compared by measuring the content of unreacted free amine groups in the sample and subsequent calculation by using the following formula:

(1) $$\mathrm{Fixation}\;\mathrm{index}\;(\%)=\frac{\left[{\left(\text{amine-reactive}\right)}_{\mathrm{fresh}}-{\left(\text{amine-reactive}\right)}_{\mathrm{fixed}}\right]}{{\left(\text{amine-reactive}\right)}_{\mathrm{fresh}}}\times 100\%$$

(amine-reactive)_fresh_: the free amine group content before crosslinking
(amine-reactive)_fixed_: the free amine group content before and after sterilization

#### 2.5.3. Differences in Water Absorption and Degradation Rate after Sterilization

A water absorption test was performed to determine the swelling ratio or water content in the hydrogel macrospheres of HyS-w/γ and HyS-w/o γ. Each group of hydrogel macrospheres (*W*_0_) was weighed and immersed in double-distilled (DD) water at 37 °C for 5, 10, 15, and 30 min as well as 1, 2, 4, 6, 8, and 24 h. The samples were taken out and rinsed, and the excess water was removed from the surfaces. The weights of the hydrated specimens (*W_wa_*) were immediately measured and compared with the weights of the dried porous spheres (*W_wa_*) (*n* = 3). The formula for measuring the water absorption of the hydrogel macrosphere is as follows:

Water absorption = (*W_wa_* − *W_o_*)*/W_o_* (2)

The degradation rate was measured by immersing the macrospheres in DD water for 1 day, weighing the standard hydrate saturation of the macrospheres (*W_h_*), and again immersing the macrospheres in DD water at 37 °C for 1–13 d. After removing the test piece, the excess water was removed from the surface. The residual weight (*W_r_*) was measured using a precision balance after water absorption. The following formula was used to calculate the degradation rate of each group of macrospheres after different periods:

Degradation rate = *(W_h_* − *W_r_)/W_h_* × 100% (3)

#### 2.5.4. Sterility Assurance, Antibacterial Ability, and Cell Viability of Hydrogel Macrospheres with Drugs 

##### Sterility Assurance

The sterility of any product is defined by the viability of a microorganism on the product after sterilization. Negative control (NC), positive control (PC), and experimental groups are essential for selecting the appropriate sterility assurance of medical devices. The NC group was sterile deionized water mixed with a liquid medium. The PC group was bacteria-containing water mixed with a liquid medium, which became turbid after 1 day of cultivation. The culture environment was heated to 35 °C, and the culture was cultivated without carbon dioxide to prevent the bacteria from growing excessively fast. The experimental group was sterilized gel macrospheres soaked in a hydrogel: culture solution with a ratio of 0.1 g:2.5 mL. Finally, the supernatant and agar culture were examined to observe whether agar colonies were formed after 1 day of cultivation.

##### In Vitro Cytotoxicity Following ISO 10993-5:2009

For the biological evaluation of the macrospheres, cytotoxicity tests were conducted following ISO 10993-5:2009. The newborn mouse fibroblast cell line of L929 was used in the aforementioned tests. The used cells were cultured with an alpha-modified Eagle’s medium (α-MEM, Gibco^®^, Life technologies Co., NY, USA) containing house serum (Biolegend Co., South Logan, UT, USA) in an incubator containing 5% CO_2_ at 37 °C. The medium was replaced once every 2 days. When the cell cultures grew to 80% capacity, they were subcultured. The PC was 15% dimethyl sulfoxide (DMSO), with each 1 mL of cell culture medium containing 150 μL of DMSO filtered through a 0.22-μm filter membrane. The NC was high-density polyethylene (HDPE) with a weight-to-medium volume ratio (g/mL) of 1:10. The medium was placed in a water bath at 37 °C for 24 h, and the extract of HDPE was set as the NC. The control group used the normal cell culture medium to cultivate the L929 cell line.

For each group, six replicate tests were measured (*n* = 6). The extraction solution was prepared by placing the irradiated macrospheres in the cell culture medium with a weight-to-medium volume ratio (g/mL) of 1:10 and heating it to 37 °C. After 24 h of incubation, the resultant supernatant liquid was the extract of the experimental group. The cell concentration of 1 × 10^4^ cells/well was transferred into a 96-well microliter plate and cultured overnight in an incubator; the original medium was aspirated, and the sample extract (100 μL/well) was added to cultivate the cells. After culturing for 24 h, the aspirated cell culture medium was used for the experimental group. A general cell culture medium (100 μL/well) was then added and mixed with a cell proliferation assay kit (XTT; 50 μL/well). The mixture was then placed in a 5% CO_2_ incubator at 37 °C for 4 h. Subsequently, the OD_490_ was measured using an ELISA reader (the XTT assay OD is proportional to the cell activity), and the cell morphology was observed.

##### Bacterial Endotoxin Testing

First, the test sample extract was prepared by adding 1 mL of pyrogen-free water to each 0.04-g batch of macrospheres and conducting ultrasonic-assisted shaking for 1 min. The extract was then placed at room temperature for 1 h before shaking again for 1 min. Then, a serial amount of pyrogen-free water was used for dilution. The NC group was prepared with 100 μL of pyrogen-free water, and the PC group was prepared using 50 μL of the 4λ endotoxin standard and 50 μL of pyrogen-free water. The positive product control group comprised 50 μL of undiluted test sample extract and 50 μL of the 4λ endotoxin standard.

Second, the sample group was prepared by diluting 50 μL of undiluted extract into 2×, 4×, 8×, and 16× dilutions by using pyrogen-free water. A volume of 100 μL of limulus amoebocyte lysate endotoxin reagent was prepared for cleaning the validation and sample groups with a reagent concentration of 0.25 endotoxin units (EU)/mL. The cleaning was conducted at 37 °C for 1 h. The standard tube agglutination test was performed; however, no agglutination was observed in the NC group.

##### Antibacterial Activity

An agar diffusion test was performed to measure the degree of antibacterial activity. The medium was tryptic soy agar, and the strains were Staphylococcus aureus (*S. aureus*) and Escherichia coli (*E. coli*). The antibiotic-loaded sterilized macrospheres were attached to the agar culture plate coated with bacteria, and the inhibition zone sizes were observed after incubating at 37 °C for 24 h. The quantitative inhibition sample was tested with the broth dilution method. The sample was immersed in 1 mL of bacterial suspension with a cell density at an OD_595_ value of 0.2 and incubated at 37 °C for 1, 4, 8, and 24 h. The control sterilized control, and experimental groups were measured after removing the bacterial liquid at each time point and measuring the OD_595_ value with an ELISA reader.

### 2.6. Statistical Analysis 

IBM SPSS was used to determine characteristics such as average pore diameter, deviation rate, and degree of crosslinking and overlap. The experimental data were statistically analyzed, and the experimental data of the two groups were compared using the t-test. Moreover, Tukey’s test was used to analyze the comparison of the groups.

## 3. Results and Discussion

### 3.1. Changes in the Physiochemical Properties of Hydrogel Macrospheres after Sterilization

**Figure 1:**
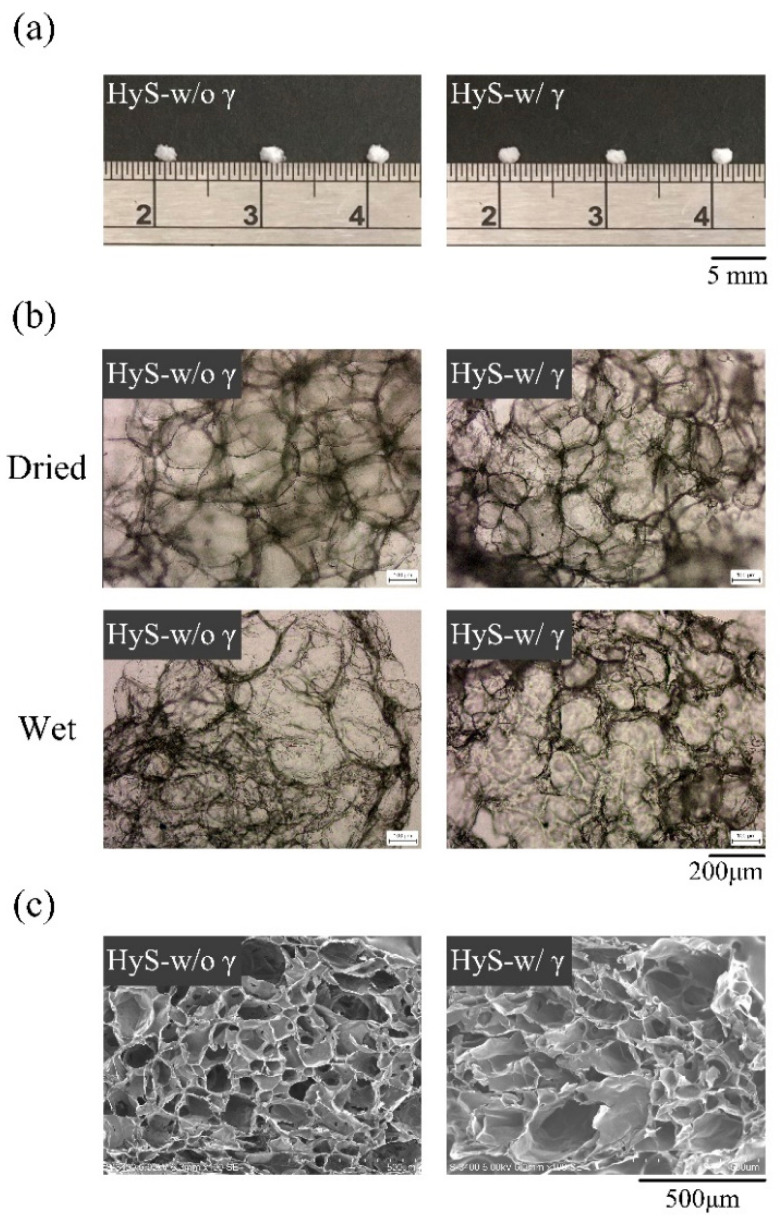
Freeze-dried hydrogel macrospheres after (HyS-w/γ) and before (HyS-w/o γ) sterilization of internal macrosphere microstructures of (**a**) photographs, (**b**) OM images, and (**c**) SEM images.

The external optical and internal microstructure images of the hydrogel macrospheres are displayed in Figure 1. The size of the prepared particle macrospheres was approximately 2 mm, and no noticeable effects on the appearance and size of the macrospheres were observed after sterilization with γ-ray irradiation (Figure 1a). Observations of the internal pores of the macrospheres before and after water absorption (Figure 1b) indicated that obvious pores appeared regardless of whether the spheres had been sterilized.

**Figure 2:**
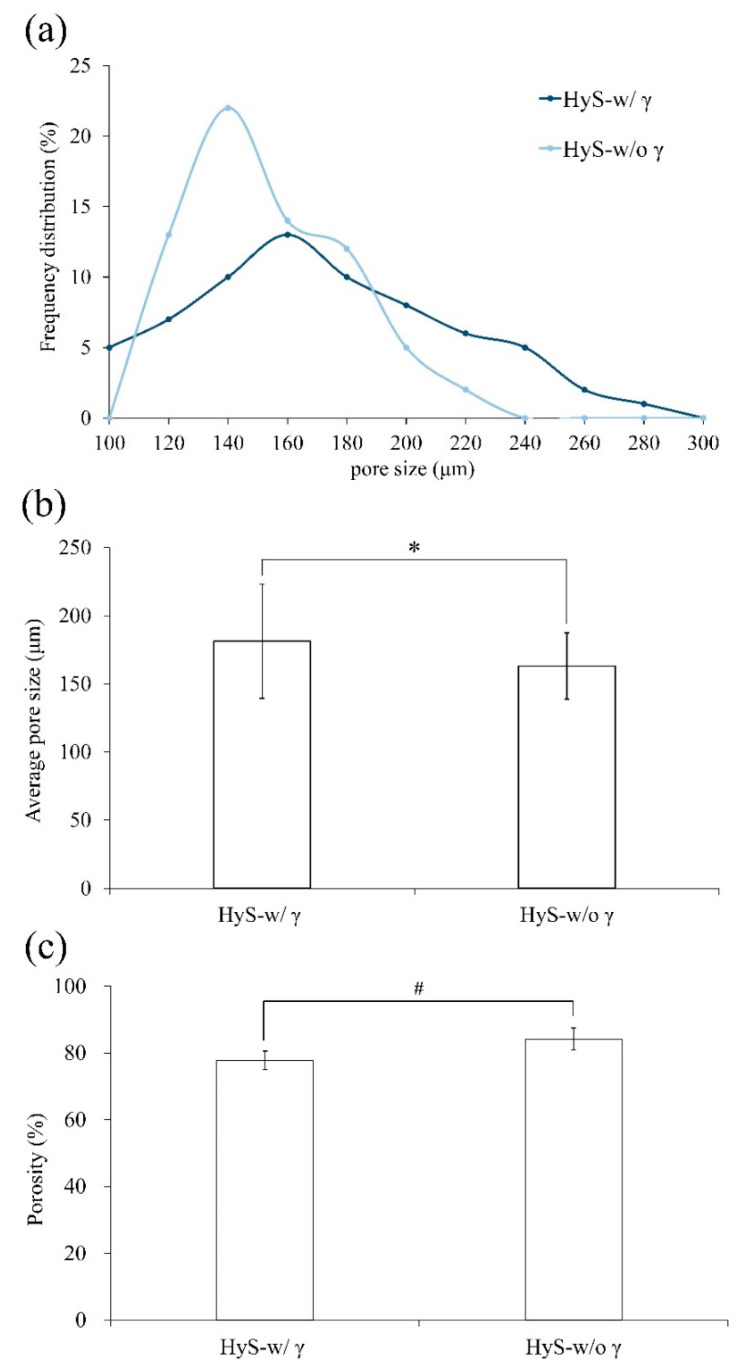
Comparisons of the (**a**) pore size distribution, (**b**) average pore size (*n* = 60; *: *p* < 0.05), and (**c**) porosity of hydrogel macrospheres before (HyS-w/o γ) and after (HyS-w/γ) sterilization (*n* = 60; #: *p* > 0.05).

This observation confirms that after sterilization, the hydrogel macrospheres retained their original pore structures (Figure 1b,c). Notably, the largest measured frequency of the internal pore diameter distribution in the hydrogel macrospheres shifted to the right, which indicated that the pores increased in size after sterilization (Figure 2a,b), and the significant difference was observed after statistical analysis (*p* < 0.05). The porosity changes in the macrospheres before and after sterilization were further analyzed using the SPSS software, which indicated that although the porosity of the macrospheres decreased marginally after sterilization (Figure 2c), no significant difference was observed after further statistical analysis (*p* > 0.05). The preliminary conclusion is that the hydrogel macrospheres maintained a uniform pore structure before and after sterilization with γ-ray irradiation.

**Figure 3:**
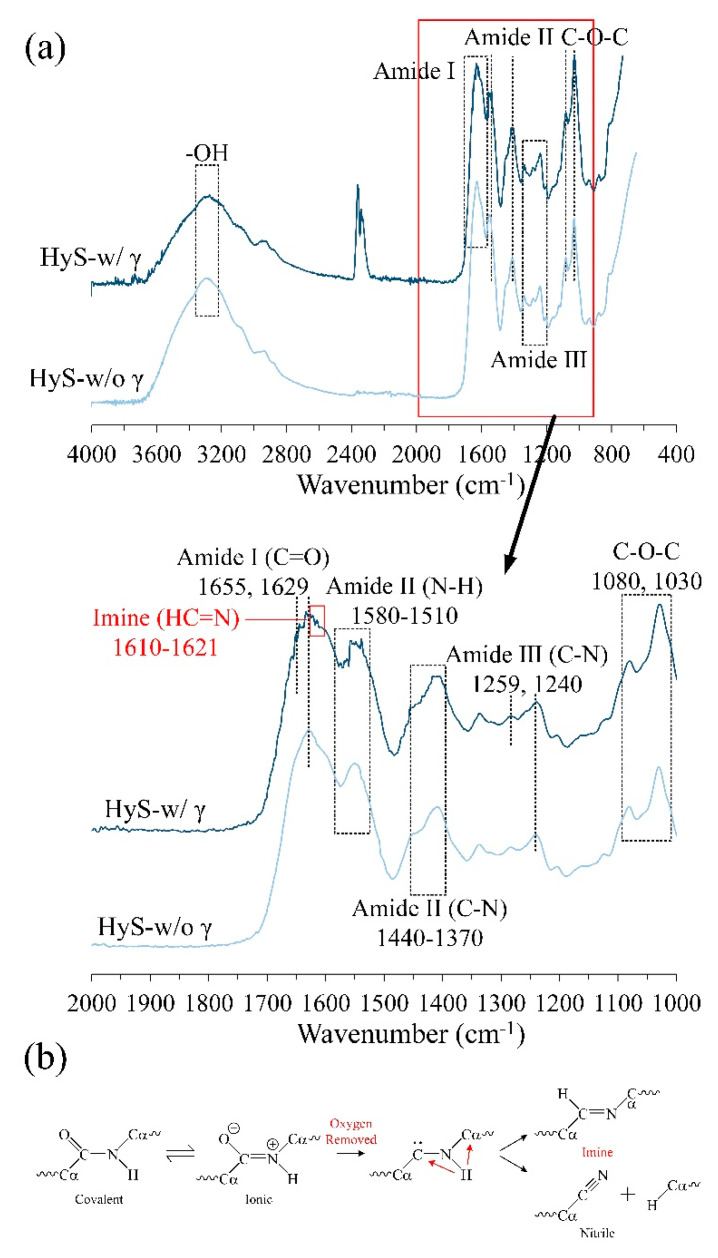
(**a**) Full and specific absorption bands obtained through the FTIR spectrum analysis of macrospheres before (HyS-w/o γ) and after (HyS-w/γ) sterilization. (**b**) Schematic of the mechanisms of amide bond decomposition into active imine or nitrile groups after irradiation, which can generate new intermolecular crosslinking sites in hydrogels.

The Fourier-transform infrared (FTIR) spectroscopy analysis results of the hydrogel macrospheres before and after sterilization are presented in Figure 3a. The characteristic peaks are as follows: the bands at 1080 and 1030 cm^−1^ for the COC group correspond to sodium alginate [50]; the vibrations at 1259 and 1240 cm^−1^ indicate amide III; the band regions at 1370–1440 and 1510–1580 cm^−1^ indicate amide II [51]; and the bands at 1655, 1629, and 1600 cm^−1^ indicate amide I of gelatin [52]. Notably, a new functional imine group (HC=N) was discovered in the band of 1610–1621 cm^−1^ for the sterilized HyS-w/γ macrospheres. [53,54] A study indicated that the amide group may decompose and transform into a new imine or nitrile group after irradiation [55]. Accordingly, the possible mechanisms of amide decomposition into more active imine or nitrile groups are displayed in Figure 3b. The amides between hydrogels decomposed immediately after irradiation and these active sites provided new sites for intermolecular recrosslinking to form a 3D interlocking structure. This process may have induced the internal pore size fluctuation and the following analysis results.

**Figure 4:**
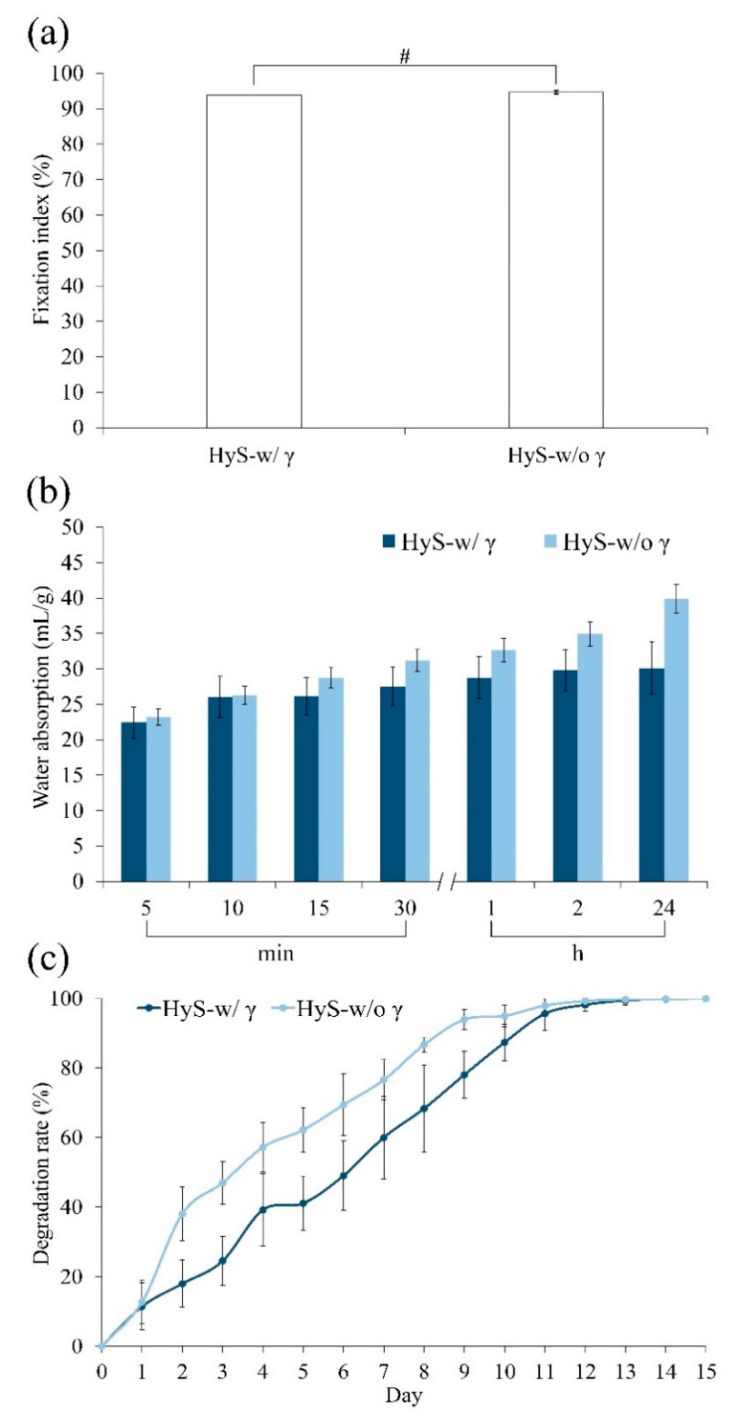
Physiochemical analysis of hydrogel macrospheres before (HyS-w/o γ) and after (HyS-w/γ) sterilization corresponds to amine fixation index (*n* = 6, #: *p* > 0.05) (**a**), water absorption ratio (*n* = 10) (**b**), and degradation rate (*n* = 10) (**c**).

The fixation index of the internal crosslinking degree of the hydrogel was directly proportional to the amount of residual free amine (Figure 4a). When the hydrogel reacted with EDC, the free amine of gelatin and carbonyl in hyaluronic acid reacted when EDC was added to produce a stable amide bond. The fixed index of crosslinking hydrogels was evaluated according to the opposite quantity of the residual amine group. Accordingly, the irradiation did not significantly affect the crosslinking degree between the hydrogels; however, γ-ray irradiation caused amide bond breakage, which exposed certain active amino groups. However, in situ crosslinking occurred again immediately; thus, the measured differences in the crosslinking degree between the groups before and after irradiation (HyS-w/o γ and HyS-w/γ) were limited.

The water absorption test results were noticeably different (Figure 4b). The degree of water absorption observed in the HyS-w/γ group plateaued from 10 min to 2 h. Subsequently, the degree of water adsorption marginally increased, and finally, macrospheres appeared after 24 h of immersion. The absorption rate reached approximately 30 mL/g. However, the degree of water absorption in the HyS-w/o γ group continued to increase until 24 h elapsed, ultimately reaching approximately 40 mL/g. The water absorption rate of the macrospheres between the HyS-w/o γ and HyS-w/γ groups significantly differed after 15 min of immersion. We speculate that the difference observed between the HyS-w/o γ and HyS-w/γ groups might have occurred because certain hydrophilic bonds crosslinked with the active imine, which resulted in the strengthening of the intermolecular force and the significant reduction of the water absorption rate of HyS-w/γ after irradiation.

The in vitro degradations of hydrogels indicate that the HyS-w/o γ group degraded faster than the sterilized HyS-w/γ group within 10 days of immersion (Figure 4c); however, both groups of immersed macrospheres were completely disintegrated after 13 days. This phenomenon confirms the hypothesis that most of the internally active groups exposed on the pore surfaces bonded with one other at an accelerated rate due to irradiation, which exacerbated the reduction in water absorption and contributed to the delay in hydrogel degradation.

Sterilization can be achieved by a combination of heat, chemicals, irradiation, high pressure, and filtration like steam under pressure, dry heat, ultraviolet, radiation, gas vapor sterilants, chlorine dioxide gas, etc. [43,44,45,46,47]. In this study, hydrogels with high viscosity were used in the design and manufacturing of the final product on the scale of macrosphere. The sterilization method of filtration is suitable for thermolabile solutions that can pass through sterile bacteria-retaining filters. However, hydrogels with high viscosity cannot pass through sterile bacteria-retaining filters. The sterilization method of heat, e.g., steam under pressure and dry heat destroys the amino, amide, or hydroxy groups in hydrogel macrospheres, and are not considerably thermal stable for our study. Therefore, the sterilization method of heat is still unsuitable [47].

**Figure A1:**
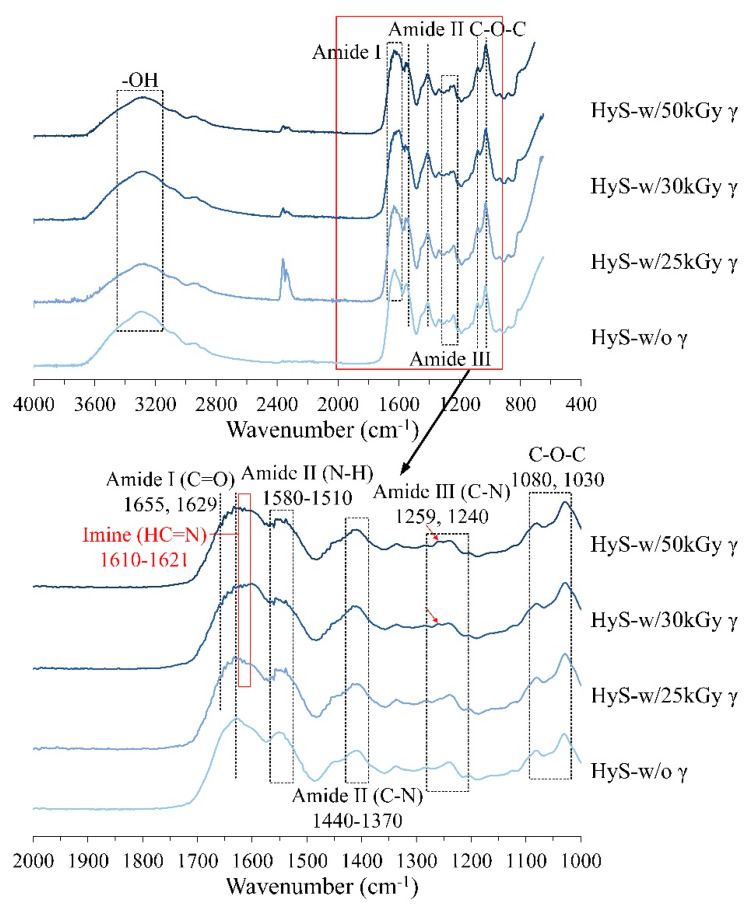
Full and specific absorption bands were obtained through the ATR spectrum analysis of macrospheres before (HyS-w/o γ) and different γ-radiation kGy after (HyS-w/γ) sterilization.

The sterilization method of chemical or gas sterilization is done with ethylene oxide or other highly volatile substances. Due to the porous macrospheres created by hydrogels that have high water or gas adsorption efficiency and stability, there is the possible issue of toxic residues remaining intact within the hydrogels in the macrospheres. Hence, gas sterilization is also not suitable here. The reviewed literature suggest that for the sterilization of hydrogels (excluding irradiation sterilization), gas sterilization or aseptic process are suitable and more credible compared to other methods [47]. Aseptic processing is conducted only for products that cannot be sterilized in final containers. Accordingly, raw materials and products of aseptic processing that have been sterilized by one of the above sterilization processes are transferred to pre-sterilized containers and sealed; both operations need to be carried out under controlled aseptic conditions. However, the whole process will cost more and the risk is higher than with irradiation sterilization. Therefore, the aseptic material filling process is suggested only if the final commercial products cannot be sterilized in the final containers. The sterilization method of irradiation, especially γ-irradiation, has a high level of penetration, is isothermal, and offers good assurance of product sterility, no chemical residue, and immediate availability of the product after sterilization. Thus, it is suitable for hydrogel macrospheres. The effect of γ-ray doses (25, 30, 50 kGy) on hydrogels is the reason why this research focused on 25 kGy (provided in Appendix A (Figure A1)).

### 3.2. Sterility Assurance as Well as Protein and Antibiotic-Releasing Abilities

**Figure 5:**
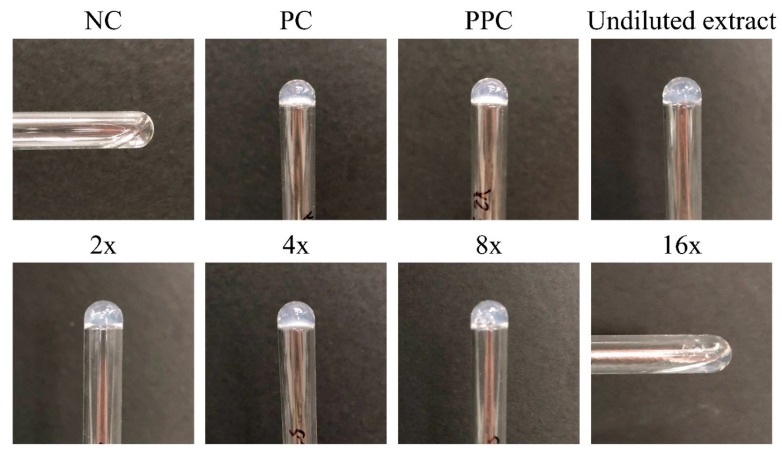
Bacterial endotoxin results of sterilized hydrogel macrospheres (NC: negative control; PC: positive control; PPC: positive product control; 2×, 4×, 8×, and 16× indicate extracts diluted with pyrogen-free water).

**Figure A2:**
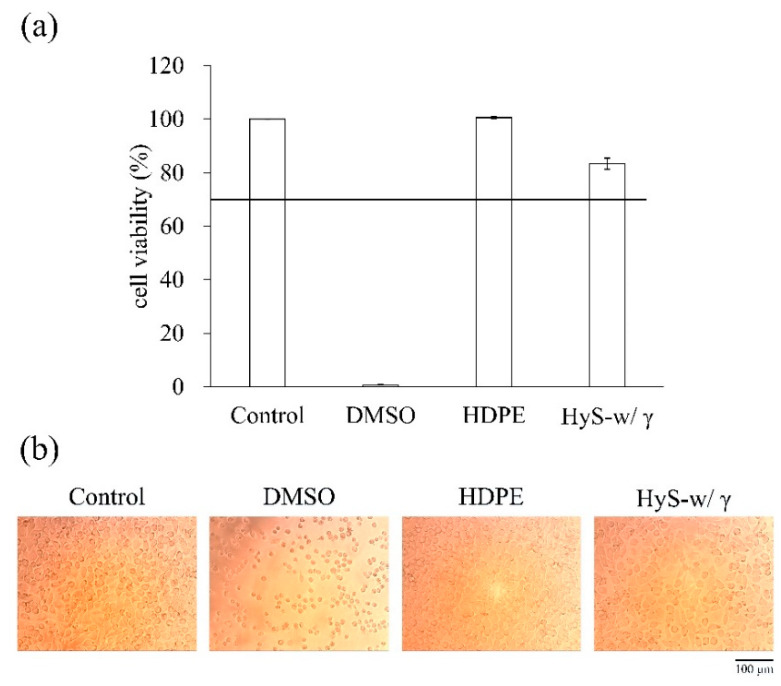
(**a**) Quantitative and (**b**) qualitative (*n* = 6) cytotoxicity test results of hydrogel macrosphere extract cultured with L929 cells for 24 h.

The endotoxin test was based on a simple gel-clot method for detecting and semiquantifying endotoxins according to lysate clotting. If the sample endotoxin contained more than 0.25 EU/mL, the reagent gel clotted. According to the ANSI/AAMI ST72 standard, the endotoxin content of each medical device package should be less than 20 EU/mL, so that it meets the requirements of pyrogen-free labware and the reagent preparation environment. The endotoxin test consisted of three control groups and five experimental groups of undiluted and diluted extracts under different magnifications to determine whether they would gel-clot. The EU concentration per mL was then reversed and converted for the hydrogels. The test results revealed that the undiluted, 2-, 4-, and 8-fold diluted reagent groups gel-clotted; however, the 16-fold diluted reagents did not gel-clot (Figure 5), which indicated that the endotoxin content of the macrospheres was between 2 and 4 EU/mL after adjusting on the standard curve; thus, this sample was endotoxin/pyrogen-free. For sterility validation, the quantitative measurement results of L929 cell viability displayed in Figure A2a reveal that the cell survival rate of HyS-w/γ was 83%, indicating that the hydrogel macrospheres were not cytotoxic after sterilization according to EN ISO 10993-5 Clause 4.1. The additional qualitative results of the L929 cells displayed in Figure A2b further validate that the HyS-w/γ process did not affect cell phenotype and growth after 24 h of cell culture.

**Figure 6:**
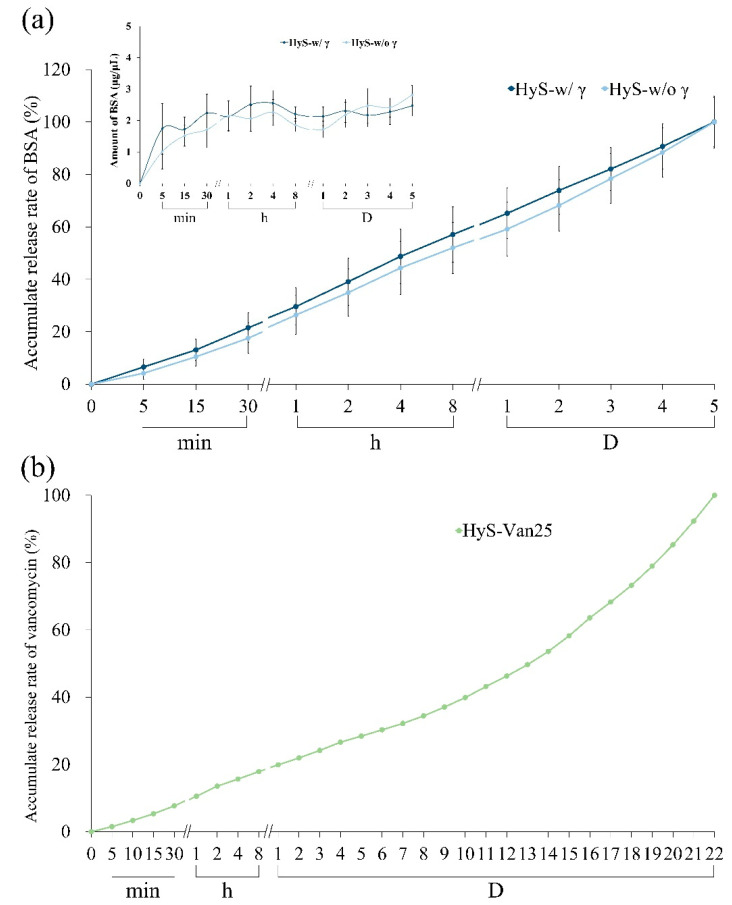
Protein release of BSA before (HyS-w/o γ) and after (HyS-w/γ) sterilization of hydrogel macrospheres in vitro (*n* = 10) (**a**) and cumulative antibiotic release of hydrogel macrospheres impregnated with vancomycin after sterilization (HyS-Van25, *n* = 10) (**b**).

To confirm whether the irradiation would stimulate a reaction between the hydrogels and the carrying additives, we measured the release ability of the hydrogel macrospheres impregnated with protein and antibiotics before and after sterilization. The curves in Figure 6a indicate the BSA releases of HyS-w/γ and HyS-w/o γ at different time points after immersion. Both groups demonstrated no considerable variations in BSA releases after 1-h tests. The release rate of HyS-w/γ marginally increased after 1 h, and the average BSA release in the microspheres was approximately 2.14 g/L. The BSA releases tended to plateau after 4 h of macrosphere immersion, and the BSA release amount was 2.47 g/L after 5 d, which indicated that the HyS-w/γ macrospheres maintained its release capability after γ-ray irradiation. 

We also examined the hydrogels impregnated with vancomycin and the accumulated release amounts of immersed macrospheres after sterilization (HyS-Van25) in DD water after 22 days of immersion (Figure 6b). The cumulative releases reached 19% within 24 h following HyS-Van25 immersion, which continuously released for up to 20 days. Different release slopes represent different release mechanisms. The release curve can be divided into two slope values at 11 days, which correspond to the complete degradation depicted in Figure 4c. The small slope value indicates that the vancomycin release in the first stage of HyS-Van25 immersion was dominated by diffusion, and the large slope value represents the vancomycin transport process in the second stage. Diffusion and structure erosion accompanied these mechanisms, thus causing another wave of rapid release.

### 3.3. Antibacterial and Biocompatible Abilities of HyS-Van25 after Irradiation

**Figure 7:**
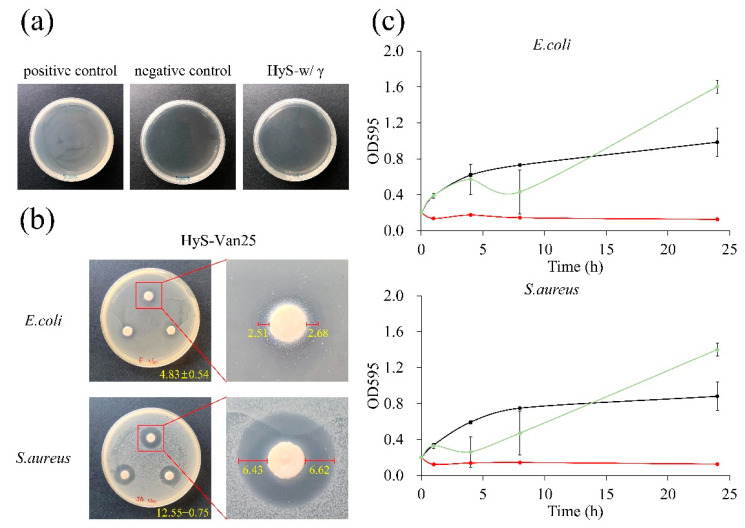
(**a**) Qualitative analysis of the sterility assurance of hydrogel macrospheres after sterilization (HyS-w/γ). (**b**) Qualitative results of the disk diffusion antibacterial effect of hydrogel macrospheres impregnated with vancomycin (HyS-Van25, *n* = 3) against *E. coli* and *S. aureus* after 24 h of interaction. Right figures indicate the inhibitory zones formed by the presence of macrospheres with a diameter of 6 mm. (**c**) Quantitative antibacterial results of hydrogel macrospheres impregnated with vancomycin after irradiation (HyS-Van25, *n* = 3) against *E. coli* and *S. aureus* after 24 h of interaction under broth dilution.

**Figure 8:**
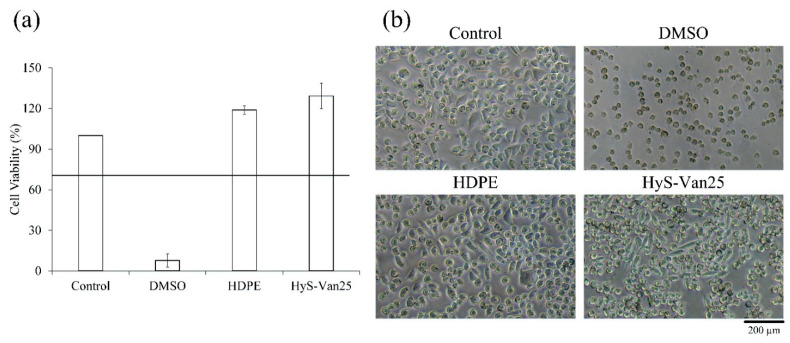
(**a**) Quantitative cell viability (*n* = 6) and (**b**) qualitative cell morphologies of L929 cells cultured for 24 h in a solution of hydrogel macrospheres impregnated with vancomycin after sterilization (HyS-Van25).

Figure 7a illustrates that after hydrogel sterility testing, compared with the contaminated PC group, the HyS-w/γ culture plate and the NC group were both completely sterile, which validated the hydrogel macrosphere sterilization procedures. The results of the qualitative and quantitative antibacterial testing of HyS-Van25 on *E. coli* and *S. aureus* are presented in Figure 7b,c, respectively. The qualitative results reveal evident antibacterial properties against both *E. coli* and *S. aureus*. According to the manufacturer’s instructions, vancomycin has excellent antibacterial ability against gram-positive bacteria, which results in better responses of HyS-Van25 against gram-positive *E. coli* than against gram-negative *S. aureus* (Figure 7c). The quantitative results of the antibacterial effects of HyS-Van25 against *E. coli* and *S. aureus* indicate that HyS-Van25 samples cultured with *S. aureus* for 4 h exhibit superior antibacterial efficiency to the same samples cultured with *E. coli* for 8 h. After 24 h of bacterial culture, the OD of HyS-Van25 was higher than that of the control group, which may have occurred because hydrogel degradation (Figure 4c) caused the solution to become turbid. Therefore, the instrument obtained a false reading. The results of the quantitative and qualitative cytotoxicity evaluations of HyS-Van25 extract cultured with the L929 cell line after 24 h are presented in Figure 8. The results revealed that even after 25-kGy γ-ray sterilization (HyS-Van25), hydrogel macrospheres were not cytotoxic (Figure 8a) and did not noticeably affect the phenotype and growth of cells (Figure 8b).

As for the ATR spectrum, when the samples are not sterilized by γ-ray and sterilized by 25-kGy irradiation, there is not much difference in the spectra (Appendix A, Figure A1). It only changes at 1610 cm^−1^, which significantly affects the water absorption ratio of hydrogel macrospheres (Figure 4b). However, after 30- and 50-kGy irradiation sterilization, a certain functional group displayed an absorption band near 1259 cm^−1^, which may affect its physical and chemical properties. Even on irradiation sterilization, each hydrogel system with and without protein or antibiotic releasing ability requires case-by-case testing to select the most suitable doses and an effective method to allow for the main properties to remain unaltered. The impact of irradiation sterilization on the physicochemical properties of the alginate/gelatin with a *w*/*w* ratio of 1/4 crosslinking gel macrospheres was understudied, and therefore further research of varying hydrogel system is needed.

## 4. Conclusions

The produced hydrogel macrospheres possessed an interconnected pore structure, and after γ-ray sterilization, the degree of crosslinking of the hydrogel macrospheres marginally reduced due to the partially decrosslinked amide in the hydrogels. This amide caused the water absorption of HyS-w/γ to decrease marginally and led to an initial degradation rate higher than that of HyS-w/o γ. However, both macrosphere groups completely degraded after 13 days of immersion. After sterilization, the hydrogel macrospheres’ ability to release BSA and vancomycin did not significantly change, which indicated that no chemical reaction occurred after 25-kGy γ-irradiation. Moreover, after loading vancomycin, the antibiotic was successfully released without cytotoxicity. The qualitative results indicated that favorable antibacterial ability remained for *E. coli* and *S. aureus*; however, the quantitative antibacterial effect on *S. aureus* was significant only when the culturing time was less than 8 h. The sterilization of the hydrogel macrospheres did not have any obvious influence on the structure of the macrospheres. However, sterilization exhibited several minor effects. According to the FTIR analysis results, a new active imine group was generated due to the breakage of the original intermolecular covalent amide bond. The experimental results indicated that macrospheres are an excellent drug or protein carrier and that the common 25-kGy dose of γ-ray irradiation used to sterilize medical devices can be used to validate hydrogel macrospheres and extended applications that can be combined with bone grafts in the future.

## Data Availability

Not applicable.

## Appendix A

For comparison, the γ-ray dose effect on hydrogels and preliminarily determined influence of the recommended 25-kGy γ-irradiation dose in this study, the results using varying doses of 25, 30, and 50-kGy γ-irradiation doses in a one-step gamma-ray sterilization process through ATR spectra are shown in Figure A1. An absorption spectra in the frequency range of the amide I band for α-helix (~1655 cm^−1^), β-sheet (~1630 cm^−1^), and random coil (~1645 cm^−1^) structures were changed into a new functional imine group (HC=N) band (1610~1621 cm^−1^) in all radiation dose groups. Besides, the new peaks from the nitrite group of amide III were observed at 1259 cm^−1^ due to the decomposition and transformation into a new imine or nitrile group of hydrogel after 30 and 50- kGy γ-irradiation doses.

For comparison, the L929 cell survival rate of the control group with a pure medium was set as 100%. The cell viability results in Figure A2a indicate that according to the ISO 10993-5 standard, the cell survival rate of the PC group with 15% DMSO was lower than 10%, which suggests that the L929 cells were toxic. The cell survival rate of HyS-w/γ was 83%, which indicates that the hydrogel macrospheres were not cytotoxic after sterilization according to EN ISO 10993-5 Clause 4.1. The qualitative results of the L929 cells displayed in Figure A2b indicate that the control group and HDPE group had normal cell-type morphologies; thus, the sterilization process was validated. In the PC group, the cells were spherical, which indicated a state of apoptosis in the L929 cells. The cell morphology of the HyS-w/γ group was similar to that of the control group without apoptosis, which indicates that the sample did not cause cell apoptosis or death.
